# Effects and mechanism of the timing of alfalfa hay supplementation on rumen development of Hu lambs

**DOI:** 10.1093/jas/skaf227

**Published:** 2025-08-23

**Authors:** Kenan Li, Haidong Du, Wenliang Guo, Meila Na, Renhua Na

**Affiliations:** College of Animal Science, Inner Mongolia Agricultural University, Hohhot 010018, China; Institute of Grassland Research of Chinese Academy of Agricultural Sciences, Hohhot 010010, China; College of Animal Science, Inner Mongolia Agricultural University, Hohhot 010018, China; College of Animal Science, Inner Mongolia Agricultural University, Hohhot 010018, China; College of Animal Science, Inner Mongolia Agricultural University, Hohhot 010018, China; College of Animal Science, Inner Mongolia Agricultural University, Hohhot 010018, China

**Keywords:** alfalfa hay supplementation time, lambs, rumen epithelial transcriptomic, rumen morphology, microbial composition

## Abstract

The present study investigated the effects of alfalfa hay supplementation at different time points on rumen development in preweaning lambs and its underlying mechanisms. Thirty-six 7-d-old lambs (3.88 ± 0.92 kg) were randomly allocated to two feeding treatments, with 18 lambs in each group. Three lambs were housed together in the same pen as a single unit. After 7 d of adaptation to the milk replacer (**MR**), one group received MR and starter pellets, along with chopped alfalfa starting at 14 d of age (early alfalfa feeding group [**EAF**]), and the other group received the same diet but with chopped alfalfa introduced at 42 d of age (late alfalfa feeding group [**LAF**]). All lambs had ad libitum access to starter pellets and chopped alfalfa. At 42, 56, and 70 d of age, six lambs were randomly selected from each treatment group for slaughter. Rumen contents and epithelial tissues were collected for bacterial 16S rRNA sequencing and transcriptome analysis, respectively. At 42 d of age, the results indicated that the body weight (*P* < 0.05), average daily gain (*P* < 0.05), total dry matter intake (*P* < 0.05), rumen papillae length (*P* = 0.04), and rumen muscle layer thickness (*P* = 0.01) were higher in the EAF compared with LAF group. Nevertheless, the α-amylase activity (*P* = 0.04) and stratum corneum thickness (*P* < 0.01) were lower in the EAF compared with LAF group. However, no statistically significant differences were observed in growth performance, rumen fermentation parameters, and tissue morphology indicators at 56 and 70 d of age between EAF and LAF groups (*P* > 0.05). The EAF group exhibited lower abundances of *Lachnospiraceae_NK3A20* and *Muribaculaceae* compared to the LAF group at 42 d of age (*P* < 0.05). The transcriptomic results showed that early supplementation with alfalfa upregulates the expression of solute carrier family 2 like 14 (***BCL2L14***) and downregulates the expression levels of keratin 2 (***KRT2***) and stearoyl-CoA desaturase (***SCD***) in 42-d-old lambs. In conclusion, supplementing alfalfa at 14 d of age is beneficial to rumen development. However, this advantage gradually diminishes as the lambs grow older. The mechanism underlying the promotion of rumen development may be related to the expression of *KRT2* and *BCL2L14*.

## Teaser

Compared with providing alfalfa hay at 42 d of age, initiating the supplementation of alfalfa at 14 d of age could promote rumen development in preweaning lambs. However, this advantage gradually diminishes as the lambs grow older. Early addition of alfalfa promotes rumen development through the regulation of keratin 2 and solute carrier family 2 like 14 gene expression.

## Introduction

For ruminants, the rumen is one of the crucial sites for digestion, absorption, and metabolism of nutrients. It is widely accepted that neonatal ruminants have a nonfunctional rumen initially, with a digestive system resembling that of monogastric animals. However, as they age, neonatal ruminants undergo a rapid transition from a milk-centric diet to one based on solid feed, accompanied by significant alterations in the structure and function of the rumen. Several studies have attempted to manipulate the rumen microbial community and rumen fermentation in young ruminants by dietary changes (Dias et al., 2017; Yang et al., 2018), rumen fluid transplantation (Yu et al., 2020), or feeding plant extracts (Ma et al., 2020) and probiotics (Chaucheyras-Durand et al., 2001), with the aim of improving rumen development.

It is generally believed that solid feed is vital for rumen development in young ruminants. Solid feed sources can be broadly classified into two categories: concentrate feed and forage. Concentrate feed, which is high in fermentable carbohydrates, after fermentation in the rumen, produces substantial amounts of propionic acid and butyric acid. This, in turn, fosters the growth of rumen epithelial papillae (Sander et al., 1959). Conversely, forage is characterized by a lower energy concentration compared to concentrate feed (Hill et al., 2008). In addition, the establishment of microbial communities with fiber-degrading functions in the rumen of preweaning ruminants is not yet complete. Therefore, early research does not recommend feeding forage before weaning (Drackley, 2008). However, feeding concentrate feeds only to preweaning ruminants can readily lead to symptoms such as incomplete keratinization and adherence to ruminal papillae (Beharka et al., 1998; Yang et al., 2015). Yang et al. (2015) and Nemati et al. (2016) have reported that feeding alfalfa or oat hay on the basis of the starter feed may improve rumen development in preweaning ruminants. However, the question of when is the best time to introduce forage, particularly for preweaning lambs, remains unanswered. Soltani et al. (2017) used 10% alfalfa hay for lambs when they were 3 d old, which increased ruminal pH but failed to improve gain of lambs. A small number of studies on calves indicate that the most suitable time to introduce hay may be from the second week of life. Providing hay too early, specifically during the first week of age, may negatively affect the calf’s nutrient digestibility and intake, as suggested by Xiao et al. (2023). Conversely, delaying hay feeding until the sixth week of age can result in decreased dry matter intake (**DMI**) and growth performance, as reported by Lin et al. (2018) and Hosseini et al. (2016). Compared with calves, the gastrointestinal tract volume of lambs is smaller, so the optimal forage supplementation time still needs further investigation. Therefore, further studies are needed to fully understand the impact of supplementing alfalfa hay at different time points on rumen morphological structure, microbiota composition, and transcriptome changes in the rumen epithelium. Our hypothesis was that introducing alfalfa supplementation at 14 d of age would enhance rumen development. The purpose of this study is to reveal the effects of alfalfa hay supplementation at different time points (14 d of age vs. 42 d of age) on rumen development in preweaning lambs and its underlying mechanisms.

## Materials and Methods

### Animal trial and feeding management

All animal procedures adhered to the National Standard of the People’s Republic of China for ethical review of animal welfare, specifically the “Laboratory Animal Guideline” (GB/T 35892-2018). The handling and utilization of animals were in strict compliance with local laws and guidelines related to animal welfare.

Thirty-six 7-d-old male Hu lambs with an average body weight (**BW**) of 3.88 ± 0.92 kg were randomly allocated to two feeding treatments, with 18 lambs in each group. Following a 7-d period of adjustment to their new environment and milk replacer (**MR**), one group received MR and starter pellets, along with chopped alfalfa starting at 14 d of age (early alfalfa feeding group [**EAF**]), and the other group received the same diet but with chopped alfalfa introduced at 42 d of age (late alfalfa feeding group [**LAF**]). The experiment lasted for 8 wk. Every three lambs were used as an experimental unit and were housed in a separate pen measuring 1.6 m × 1.6 m. Thus, there are six pens in each treatment group. Inside each pen, there are three buckets, one for starter pellets, one for alfalfa hay, and one for water. The lambs had free access to starter pellets and alfalfa hay. The alfalfa hay was cut into about 2.20 cm using a hay cutter (Sida 9Z-4C type, Luoyang Sida Agricultural Machinery Co., Ltd., Luoyang, China). Supplementary Table S1 displays the nutrient levels for MR, starter pellets, and alfalfa hay. The lambs were weighed before morning feeding every week. From day 14 to day 70 of age, the daily intake of starter pellets and alfalfa hay was documented by noting the amounts offered and refused. Following this, the **DMI** and average daily gain (**ADG**) were computed based on these recordings.

Each lamb was given MR through artificial feeding from 7 to 70 d of age. To stimulate their consumption of starter pellets and alfalfa hay, MR was administered at 720 mL/d (based on consumption during the adaptation period) on the first day of the first week, then gradually reduced by 40 mL/d until it reached 200 mL/d. From 7 to 28 d of age, each lamb was fed four times daily (at 08:00, 12:00, 16:00, and 20:00), and from 29 to 70 d of age, they were fed three times daily (at 8:00, 12:00, and 18:00). The MR was dissolved in hot water at a temperature of 45 to 50 °C, and offered to lambs once it reached a temperature of 38 ± 1 °C. The mass-to-volume ratio of MR to water is 1 g/7 mL.

### Sample collection and chemical analysis

Six lambs (one from each pen) were randomly chosen and sacrificed for each treatment group. After gently mixing the rumen contents with hands, the rumen wall was cut open from the dorsal sac to take out the rumen contents, which were then divided into two parts. One part of the rumen contents was loaded into 2 mL cryogenic vials, immediately immersed in liquid nitrogen and then stored at −80 °C for the extraction of microbial DNA analysis. The other part was filtered through four-layer gauze and immediately pH-measured using a pH meter (Starter 300; Ohaus Instruments Co. Ltd., Shanghai, China). Then, the filtrate was divided into three portions, loaded into centrifuge tubes, and kept at −20 °C for the assessment of volatile fatty acids (**VFAs**), cellulase, and α-amylase levels. The rumen filtrate was mixed with 25% (*w/v*) metaphosphoric acid at a 5:1 ratio (ruminal fluid to acid) and preserved at −20 °C for subsequent VFA analysis by gas chromatography (PE Clarus 680; PerkinElmer, USA) (Lin et al., 2019). The activities of cellulase (determined by enzyme colorimetry) and α-amylase (determined by iodine–starch colorimetry) in the rumen fluid were measured according to the kit methods provided by Beijing Solarbio Science & Technology Co., Ltd and Nanjing Jiancheng Bioengineering Institute Co., Ltd, respectively. Subsequently, two segments of rumen wall tissue were obtained from the ventral sac. One segment was fixed in 4% paraformaldehyde for histomorphometric microscopy analysis. Another segment was obtained by using a sterilized forceps to perform blunt dissection to remove the muscular layer, thereby exposing the rumen epithelium. Afterward, this segment was promptly rinsed three times with ice-cold phosphate-buffered saline, rapidly frozen in liquid nitrogen, and kept at −80 °C for subsequent host transcriptome sequencing.

### Measurement of rumen tissue morphology

The rumen epithelium was embedded in paraffin sections with a thickness of 6 μm after conventional alcohol dehydration. The rumen’s tissue structure was examined under a light microscope following staining with Yihong-hematoxylin. Using Image-Pro Express (Media Cybernetics, Bethesda, MD, USA), three nonconsecutive sample sections were analyzed, and predefined criteria (Sun et al., 2018)  were measured. In brief, the stratum corneum (**SC**) is the outermost, heavily stained layer without nuclei. The stratum granulosum (**SG**) is lightly stained with cells perpendicular to stratum spinosum (**SS**) and stratum basale (**SB**). SS and SB lie between the lamina propria and SG (Supplementary Figure S1).

### DNA extraction, 16S rRNA sequencing, and data analysis

Once thawed, the rumen content samples underwent DNA extraction using a commercial DNA Kit (Omega Bio-tek, Norcross, GA, USA), following the manufacturer’s instructions. The quality and concentration of the obtained DNA were analyzed using 1% agarose gel electrophoresis and a NanoDrop 2000 UV spectrophotometer (Thermo Fisher, Waltham, MA, USA). Then, we amplified the V3 to V4 region of bacterial rRNA genes using universal primers with sample-specific barcodes. The bacterial primers were 338F (5′-ACTCCTACGGGAGGCAGCAG-3′) and 806R (5′-GGACTACNNGGGTATCTAAT-3′). The PCR reaction mixture included 4 μL 5 × Fast Pfu buffer, 2 μL 2.5 mM dNTPs, 0.8 μL each primer (5 μM), 0.4 µL TransStart Fast Pfu DNA Polymerase (TransGen AP221-02 model), 10 ng of template DNA, and ddH_2_O to a final volume of 20 µL. PCR amplification steps were as follows: pre-denaturation at 95 °C for 3 min, then denaturation at 95 °C for 30 s, annealing at 55 °C for 30 s, and extension at 72 °C for 30 s, 27 cycles were carried out, followed by extension at 72 °C for 10 min. The PCR products were analyzed by 2% agarose gel electrophoresis, following which the target fragments were recovered. The recovered products were then quantified using a Qubit 4.0 (Thermo Fisher Scientific). After PCR amplification, all amplicon libraries underwent paired-end sequencing on the Illumina MiSeq PE 300 platform (Illumina, San Diego, CA, USA).

The raw data format from the sequencing machine is in Fastq. High-quality sequences were obtained after screening using QIIME (version 1.9.1) software. The quality standards during the filtration process are consistent with those in Li et al. (2024). Using UPARSE (version 11) (Edgar, 2013), sequences were clustered into operational taxonomic units (**OTUs**) based on 97% similarity for cluster analysis. During the clustering process, chimeras were removed to obtain OTU representative sequences. Using the RDP Classifier (version 11.5) software, species classification annotation was performed for each sequence by comparing it against the Silva database (version 138). After obtaining the relative abundance information table, subsequent statistical analysis was conducted. Community diversity indices include alpha and beta diversity indices. Multivariate statistical analysis was conducted to identify rumen content microbiota with significant differences between groups.

### Epithelial RNA extraction and sequencing

Total RNA was extracted from rumen epithelial tissue using TRIzol (Invitrogen, Carlsbad, CA, USA) and subsequently analyzed for concentration and purity with a NanoDrop 2000 spectrophotometer (Thermo Fisher Scientific); for integrity by 1% agarose–formaldehyde gel electrophoresis to assess RNA quality; and for the RNA integrity number (**RIN**) using an Agilent 5300 Bio-analyzer (Agilent, Santa Clara, CA, USA). RNA with RIN >6.5 was used to construct the RNA-seq library, enriched with poly-A-tailed mRNA using TrueSeq Kit (Illumina). Majorbio Bio-pharm Technology Co., Ltd (Beijing, China) sequenced the RNA libraries using the HiSeq 6000 system (Illumina, Inc.).

Using HISAT2 (http://ccb.jhu.edu/software/hisat2/index.shtml) for sequence alignment to the sheep genome (*Ovis aries* v1.0), we then calculated host gene expression in transcripts per million reads after normalization. The differentially expressed genes (**DEGs**) were performed with pairwise comparison analysis. The significant DEGs were identified at false discovery rate (**FDR**) ≤0.05 and fold change >2 (using bioinformatics tool DESeq2). The enrichment analysis of genes based on the Gene Ontology (**GO**) was conducted utilizing Goatools (version 0.6.5), while the Kyoto Encyclopedia of Genes and Genomes (**KEGG**) pathways enrichment analysis for DEGs was executed using KOBAS (version 2.1.1).

### Gene expression quantified by qRT-PCR

We selected six genes related to rumen fatty acid metabolism to validate the results of transcriptomics, including 3-hydroay-3-methylglutaryl-CoA synthase 2 (***HMGCS2***), 3-hydroxybutyrate dehydrogenase 1 (***BDH1***), acetyl-CoA acetyltransferase 1 (***ACAT1***), 3-hydroxymethyl-3-methylglutaryl-CoA lyase (***HMGCL***), solute carrier family 9 member A3 (***SLC9A3***), and solute carrier family 9 member A2 (***SLC9A2***), which were quantified using qRT-PCR. RNA extraction was the same as transcriptome measurement. The qRT-PCR procedure was carried out using a 10 μL reaction mixture comprising 5 μL of 2× SYBR Green PCR Master Mix (AG, Hunan, China) as the fluorescent indicator, 0.2 μL of each primer at a concentration of 0.2 μM, 3.6 μL of ddH_2_O, and 1 μL of DNA templates. The relative expression levels of mRNA were adjusted based on the expression of glyceraldehyde-3-phosphate dehydrogenase (**GAPDH**, a standard housekeeping gene), with the results being computed employing the 2^−ΔΔCT^ method. The primer sequencings designed and amplification conditions for this study are presented in Supplementary Table S2.

### Statistical analysis

The data on growth performance, rumen fermentation parameters, tissue morphology, and rumen epithelial gene expression of lambs from each group were analyzed by a one-way ANOVA to examine the differences between two groups or among different ages with the individual pen as experimental unit.

The data on rumen fermentation parameters and tissue morphology of lambs from each group were analyzed by a two-way ANOVA using PROC GLM of SAS (SAS Inst. Inc., Cary, NC, USA) to examine the interaction of treatment and age with the individual pen as experimental unit. The statistical model is as follows:


$$\begin{array}{l} Yij\;=\;\mu \;+\;Ti\;+\;Pj\;+\;TPij\;+\;Ak\left(Ti\right)\;+\;\varepsilon \;ijk \end{array}$$

where Yij is the dependent variable, μ is the overall mean, Ti is the fixed effect of alfalfa hay provision timing (14 d of age vs. 42 d of age), Pj is the fixed effect of age (42, 56, and 70 d of age), TPij is the interaction of treatment and age, Ak(Ti) is the random effect of animal within treatment (k = 1 to 6), and εijk is the error term.

Multiple comparisons of means among treatments were performed using Tukey’s multiple-range tests. Data means significance is declared at *P* < 0.05.

## Results

### Feed intake and growth performance

**Figure 1.**
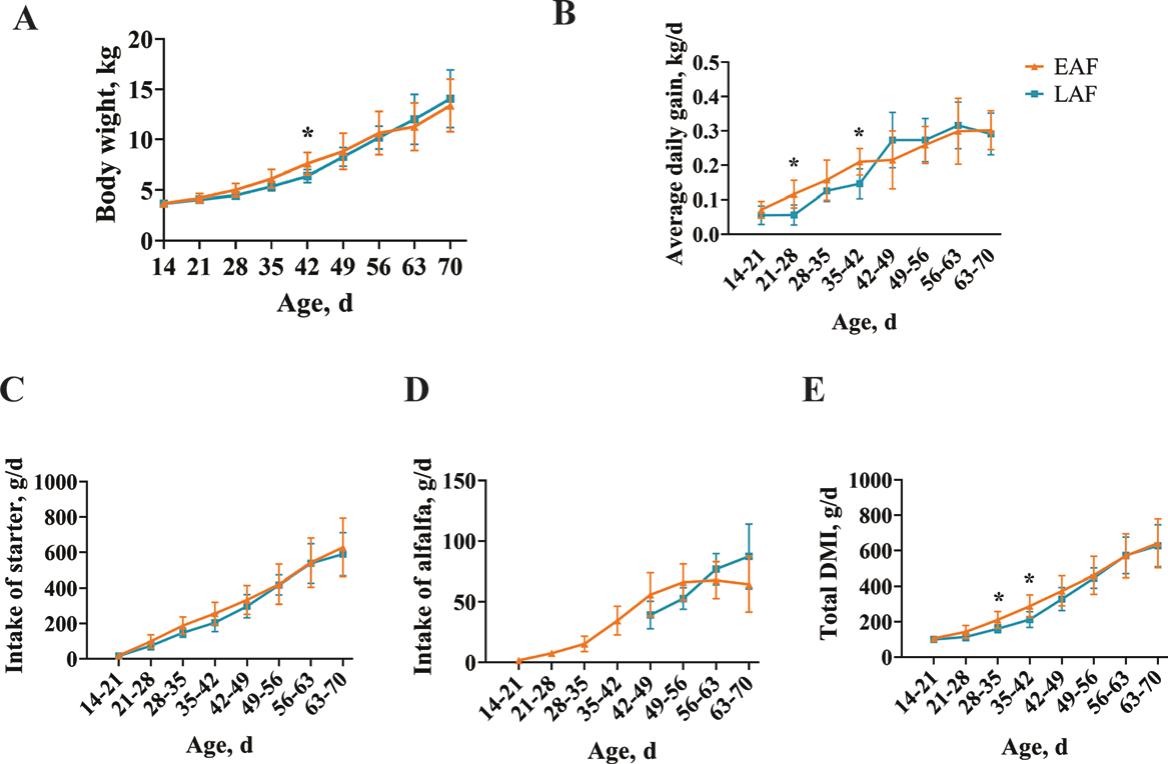
BW (A), ADG (B), starter intake (C), alfalfa hay intake (D), and total DMI (E) of lambs fed alfalfa hay at 14 d of age (early alfalfa hay feeding group [EAF]) or at 42 d of age (late alfalfa hay feeding group [LAF]). Total DMI includes MR, starter, and alfalfa hay. Asterisks (*) indicate differences (*P* < 0.05) between treatments.

As depicted in Figure 1A, at 42 d of age, the BW of lambs in the EAF group was higher than the LAF group (*P* < 0.05). However, there was no difference in BW between the EAF and LAF groups on the other days (*P* > 0.05). The ADG of lambs in the EAF group was higher than the LAF group during the periods of 21 to 28 d (*P* < 0.05) and 35 to 42 d (*P* < 0.05) (Figure 1B). No statistically significant difference was observed in starter pellet intake between the EAF and LAF groups across the age range of 14 to 70 d of age (*P* > 0.05) (Figure 1C). Surprisingly, the alfalfa hay intake had no significant difference between treatments from 42 to 70 d of age (*P* > 0.05) (Figure 1D). The total DMI of the EAF lambs aged 28 to 42 d was higher than that of LAF lambs (*P* < 0.05) (Figure 1E). However, subsequent to 42 d of age, no statistically significant difference in total DMI was observed between treatment groups (*P* > 0.05).

### Rumen fermentation parameters

As presented in Table 1, at 42 d of age, the α-amylase activity was higher in the LAF group compared to the EAF group (*P* = 0.04). However, there was no significant difference in α-amylase activity between the EAF group and the LAF group at 56 and 70 d of age (*P* > 0.05). In the EAF group, the acetate/propionate ratio in lambs was significantly higher at 42 d of age compared to that at 56 and 70 d of age (*P* = 0.04). Additionally, the α-amylase activity at 56 d of age was significantly higher than that at 42 d of age (*P* = 0.03). In the LAF group, both rumen pH (*P* < 0.01) and cellulase activity (*P* < 0.01) were significantly higher at 56 and 70 d of age compared to that at 42 d of age. Moreover, the isobutyrate concentration was significantly higher at 42 and 56 d of age compared to that at 70 d of age (*P* < 0.01).

**Table 1.** Effect of the timing of hay supply on rumen fermentation parameters

Items	Diet^1^	Days of age			*P* for diets			*P* for age	Diet * age
		42 d	56 d	70 d	42 d	56 d	70 d		
pH	EAF	6.55 ± 0.75	6.72 ± 0.63	7.07 ± 0.28	0.20	0.86	0.65	0.32	0.27
	LAF	6.03 ± 0.54^B^	6.77 ± 0.34^A^	7.13 ± 0.18^A^				<0.01	
Acetate (mmol/L)	EAF	52.64 ± 14.19	34.02 ± 8.07	49.33 ± 26.47	0.88	0.23	0.96	0.29	0.78
	LAF	50.68 ± 20.46	44.94 ± 17.10	50.21 ± 31.44				0.92	
Propionate (mmol/L)	EAF	28.11 ± 15.61	20.95 ± 6.99	30.35 ± 16.97	0.89	0.27	0.23	0.73	0.81
	LAF	30.43 ± 18.90	31.21 ± 15.20	42.73 ± 11.35				0.41	
Butyrate (mmol/L)	EAF	2.96 ± 0.16	2.26 ± 0.56	2.22 ± 0.68	0.37	0.31	0.33	0.13	0.87
	LAF	3.70 ± 1.51	2.94 ± 1.16	3.52 ± 2.39				0.77	
Isobutyrate (mmol/L)	EAF	0.39 ± 0.15	0.47 ± 0.25	0.58 ± 0.21	0.54	0.54	0.13	0.71	0.08
	LAF	0.24 ± 0.08^B^	0.40 ± 0.11^B^	1.13 ± 0.68^A^				<0.01	
Isovalerate (mmol/L)	EAF	0.21 ± 0.12	0.54 ± 0.31	0.55 ± 0.30	0.40	0.62	0.10	0.10	0.41
	LAF	0.34 ± 0.23	0.62 ± 0.15	0.95 ± 0.21				0.20	
Valerate (mmol/L)	EAF	0.74 ± 0.44	0.77 ± 0.37	1.18 ± 0.88	0.07	0.12	0.32	0.50	0.80
	LAF	1.87 ± 0.87	1.53 ± 0.92	1.79 ± 0.97				0.86	
Acetate/propionate	EAF	4.07 ± 1.87^A^	1.85 ± 0.42^B^	2.00 ± 0.46^B^	0.24	0.85	0.44	0.04	0.24
	LAF	2.39 ± 1.21	1.79 ± 0.52	1.64 ± 0.68				0.50	
Total VFA (mmol/L)	EAF	66.65 ± 20.22	61.70 ± 13.54	73.65 ± 29.14	0.79	0.17	0.89	0.76	0.98
	LAF	71.28 ± 20.09	88.90 ± 31.34	77.75 ± 37.10				0.78	
α-Amylase (U/dL)	EAF	891.16 ± 285.54^bB^	1495.50 ± 196.46^A^	1148.09 ± 476.02^AB^	0.04	0.09	0.78	0.03	0.17
	LAF	1682.17 ± 515.26^a^	1879.07 ± 289.42	1230.53 ± 250.98				0.14	
Cellulase (U/mL)	EAF	38.22 ± 21.29	38.96 ± 9.22	42.28 ± 9.65	0.13	0.15	0.40	0.88	0.52
	LAF	23.86 ± 2.80^B^	32.55 ± 3.94^A^	37.62 ± 8.70^A^				<0.01	

^1^EAF: early (at 14 d of age) feeding of alfalfa. LAF: late (at 42 d of age) feeding alfalfa.

^a–b^Within a column, means without a common superscript differ (*P* < 0.05). ^A–B^Within a row, means without a common superscript differ (*P* < 0.05).

### Histology and morphology of rumen

As presented in Table 2, at 42 d of age, the EAF group exhibited a greater rumen papillae length (*P* = 0.04) and muscle layer thickness (*P* = 0.01) compared to the LAF group. Additionally, the EAF group had a thinner SC (*P* < 0.01) than the LAF group at the same age. The rumen empty weight (*P* < 0.01), rumen papillae length (*P* < 0.05), and muscle layer thickness (*P* < 0.01) all influenced by the age in the EAF and LAF groups. In the EAF group, the total epithelia thickness was significantly higher at 70 d of age compared to that at 42 d of age (*P* = 0.03). Moreover, both the SC (*P* < 0.01) and SG (*P* = 0.02) were significantly higher at 70 d of age compared to that at 42 d of age. Furthermore, there was a significant interaction between diet and age on SC thickness (*P* < 0.01). As shown in Supplementary Figure S2B, the feed plaque was present on the rumen mucosa of the LAF group at 42 d of age, whereas it was absent in the EAF group at the same age (Supplementary Figure S2A).

**Table 2.** Effect of the timing of hay supply on rumen weight, papillae length, and the thickness of different stratum of rumen in lambs

Items	Diet^1^	Days of age			*P* for diets			*P* for age	Diet * age
		42 d	56 d	70 d	42 d	56 d	70 d		
Rumen weight									
Rumen empty weight, g	EAF	133.89 ± 21.71^B^	267.47 ± 64.42^A^	300.85 ± 82.78^A^	0.08	0.38	0.99	<0.01	0.80
	LAF	110.57 ± 20.33^C^	240.68 ± 32.21^B^	301.18 ± 68.54^A^				<0.01	
Papillae morphology									
Rumen papillae length (μm)	EAF	1398.90 ± 218.68^aB^	1657.32 ± 342.76^AB^	2007.56 ± 395.77^A^	0.04	0.70	0.70	0.02	0.40
	LAF	1031.89 ± 323.32^bB^	1543.97 ± 606.31^AB^	2100.33 ± 273.80^A^				<0.01	
Rumen papillae width (μm)	EAF	319.26 ± 21.48	323.79 ± 37.88	322.82 ± 48.58	0.20	0.96	0.84	0.98	0.69
	LAF	301.71 ± 20.34	324.73 ± 31.40	327.73 ± 27.80				0.22	
Rumen muscular layer (μm)	EAF	930.54 ± 120.06^aB^	1045.79 ± 96.41^B^	1388.60 ± 164.24^A^	0.01	0.20	0.96	<0.01	0.26
	LAF	722.38 ± 79.45^bC^	930.87 ± 181.60^B^	1393.92 ± 165.37^A^				<0.01	
Thickness of different stratum									
Total epithelia (μm)	EAF	116.59 ± 21.13^B^	135.10 ± 14.29^AB^	144.86 ± 13.33^A^	0.13	0.88	0.86	0.03	0.25
	LAF	132.66 ± 5.23	136.25 ± 10.63	143.86 ± 4.47				0.07	
SC (μm)	EAF	20.20 ± 4.05^bC^	31.12 ± 5.14^B^	37.16 ± 2.64^A^	<0.01	0.14	0.38	<0.01	<0.01
	LAF	41.05 ± 4.87^a^	35.59 ± 4.41	39.07 ± 4.40				0.14	
SG (μm)	EAF	18.47 ± 1.62^B^	19.01 ± 1.40^B^	21.72 ± 2.60^A^	0.78	0.92	0.86	0.02	0.94
	LAF	18.06 ± 2.98	19.16 ± 3.15	21.93 ± 1.38				0.06	
SS and stratum basale (μm)	EAF	77.92 ± 18.00	84.98 ± 11.79	85.99 ± 10.71	0.92	0.58	0.51	0.56	0.95
	LAF	77.10 ± 7.34	81.51 ± 9.36	82.86 ± 3.31				0.37	

^1^EAF: early (at 14 d of age) feeding of alfalfa. LAF: late (at 42 d of age) feeding alfalfa.

^a–b^Within a column, means without a common superscript differ (*P* < 0.05). ^A–B^Within a row, means without a common superscript differ (*P* < 0.05).

### Characteristics of the rumen microbiota in response to the timing of alfalfa hay supply

#### Diversity and richness of rumen microbial communities

After sequence processing, we obtained a total of 2,005,990 high-quality sequencing reads from 36 rumen samples, averaging 47,762 ± 3,625 reads per sample. Using a 97% nucleotide sequence identity threshold, we identified 2,714 OTUs, with an average of 162 ± 45 OTUs per sample.

At 42, 56, and 70 d of age, the rumen microbiota abundance and diversity in the EAF group did not differ significantly from those in the LAF group, as evidenced by the Sobs (Supplementary Figure S3A), Shannon (Supplementary Figure S3B), and Chao indices (Supplementary Figure S3C), all of which remained below the threshold for statistical significance (*P* > 0.05). Further analysis revealed changes in the alpha diversity of the rumen bacterial composition across different ages within each group. Specifically, in the EAF group, the Sobs index of rumen microbiota was higher at 56 d of age than 42 d of age (*P* < 0.05) (Supplementary Figure S3D). As shown in Supplementary Figure S3G and I, in the LAF group, both the Sobs index and Chao index were significantly higher at 70 d of age compared to 42 d of age (*P* < 0.001) and at 56 d of age (*P* < 0.05).

The principal coordinate analysis (PCoA) of the rumen microbial data was performed using both unweighted UniFrac distances (Supplementary Figure S3J). This analysis revealed significant clustering in the community structure between the EAF and LAF groups at 42 d of age (*R* = 0.355, *P* = 0.001), indicating distinct microbial profiles. Additionally, the community structure of different age groups also showed significant clustering, suggesting age-related variations in the microbial composition. However, there was no clear distinction between the EAF and LAF groups at 56 and 70 d of age, suggesting that the microbial community of these two treatment groups were similar at these time points.

#### Rumen bacterial composition and differences at different taxonomic levels

**Figure 2.**
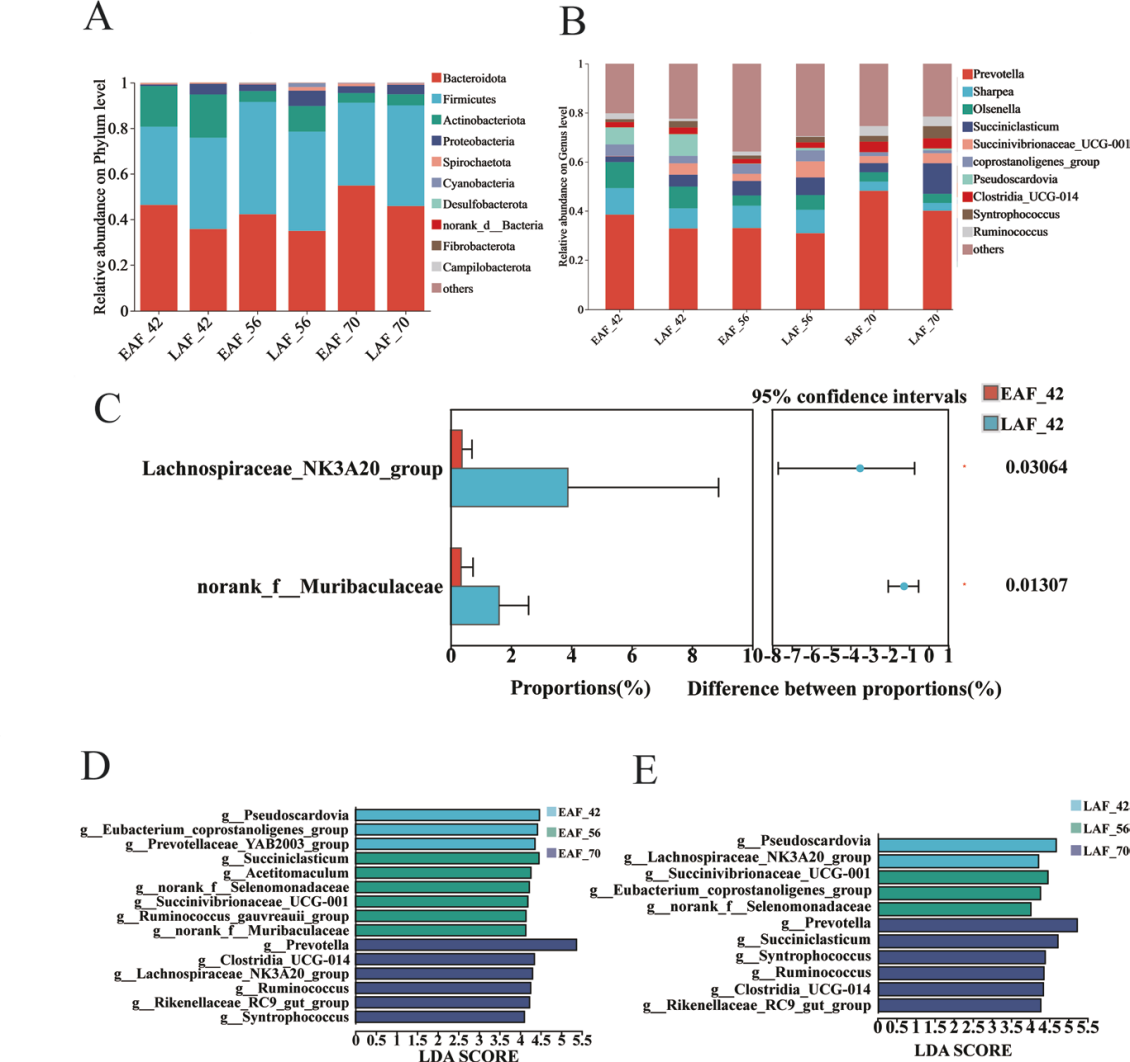
The effects of the timing of hay supply and age on the composition and differences in the rumen microbial taxa. The composition of rumen microbiota at phylum level (A) and genus level (B). Differences of rumen bacterial taxa of lambs at genus level between EAF and LAF group at 42 d of age (C). The Wilcoxon rank-sum test analysis was performed, and the FDR method was used for *P* value correction. The bacterial biomarker identified by LEfSe analysis for each age in the EAF (D) and LAF (E) groups (linear discriminant analysis [LDA] score > 4).

A total of 17 distinct phyla of rumen bacterial taxa were identified in the EAF and LAF groups. Across all samples, Bacteroidetes was the most abundance phylum, followed by Firmicutes, Actinobacteriota, and Proteobacteria. Together, these phyla comprised over 91.9% of all sequencing reads (Figure 2A).

At the genus level, a total of 333 genera were identified, with *Prevotella* being the most abundant, followed by *Sharpea*, *Olsenella*, *Succiniclasticum*, and *Succinivibrionaceae*_UCG-001 (Figure 2B). Subsequently, we employed the Wilcoxon rank-sum test to analyze the composition of rumen bacterial taxa (with a relative abundance exceeding 1.0%) in lambs, taking into account the timing of alfalfa hay supply, at specific ages (42, 56, and 70 d) during sampling. At 42 d of age, the genera *Lachnospiraceae*_*NK3A20*_group and norank_f_*Muribaculaceae* were significantly more abundant (*P* < 0.05) in the LAF group (3.88% and 1.60%, respectively) compared with the EAF group (0.38% and 0.34%, respectively) (Figure 2C). Furthermore, there were no statistically significant differences observed in the rumen microbiota composition between the EAF and LAF groups at the ages of 56 and 70 d.

To gain insights into how the timing of hay introduction influences the colonization pattern of rumen microbiota in preweaning lambs, a comparison of the temporal changes in rumen microbiota colonization between the EAF and LAF groups is essential. Using the LEfSe (linear discriminant analysis effect size) algorithm, biomarkers for each age within the EAF group’s rumen microbiota colonization process, spanning from 42 to 70 d of age, were identified. For example, *Pseudoscardovia*, *Eubacterium_coprostanoligenes*_group, and *Prevotellaceae*_*YAB2003*_group were enriched at 42 d of age; *Succiniclasticum*, *Acetitomaculum*, norank_f_*Selenomonadaceae*, *Ruminococcus*_*gauvreauii*_group, *Succinivibrionaceae*_*UCG-001*, and norank_f_*Muribaculaceae* were abundant at 56 d of age; *Prevotella*, *Clostridia*_*UCG-014*, *Lachnospiraceae*_*NK3A20*_group, *Ruminococcus*, *Rikenellaceae*_*RC9*_gut_group, and *Syntrophococcus* were enriched at 70 d of age (Figure 2D). For rumen microbiota of the LAF group, bacterial biomarkers for each age were also identified. At 42 d of age, *Pseudoscardovia* and *Lachnospiraceae*_*NK3A20*_group were enriched in the ruminal community. At 56 d of age, *Succinivibrionaceae*_UCG-001, *Eubacterium*_*coprostanoligenes*_group, and norank_f_*Selenomonadaceae* were the abundant genera. At 70 d of age, the enriched genera include *Prevotella*, *Succiniclasticum*, *Syntrophococcus*, *Ruminococcus*, *Clostridia*_*UCG-014*, and *Rikenellaceae*_*RC9*_gut_group (Figure 2E).

### Transcriptome profiling analysis

#### Differential gene expression analysis

From 36 rumen epithelium samples, a total of 1,866.80 million high-quality paired reads were obtained, averaging at 51.86 ± 7.38 million reads per sample. The percentage of reads aligned to the *Ovis aries* genome was 96.42% ± 0.62%. To investigate the effect of the timing of hay supply on the transcription reprogramming of rumen epithelial in preweaning lambs, we compared the differences in gene expression between the EAF and LAF groups at 42, 56, and 70 d of age. Additionally, we examined the temporal dynamics of the rumen epithelium transcriptome in response to different feeding scheme (Supplementary Figure S4). Among the encoded genes, there were three DEGs identified between the EAF and LAF groups at 42 d of age, including one upregulated gene (solute carrier family 2 like 14 [***BCL2L14***]) and two downregulated genes (keratin 2 [***KRT2***] and stearoyl-CoA desaturase [***SCD***]). Only one (CUB and Sushi multiple domains 2, ***CSMD2***) upregulated DEG was identified between EAF and LAF at 56 d of age, and one (synuclein alpha, ***SNCA***) downregulated DEG was identified at 70 d of age. In the EAF group, 7, 238, and 25 DEGs were identified between 42 d of age vs. 56 d of age, 42 d of age vs. 70 d of age, and 56 d of age vs. 70 d of age, respectively. Among these DEGs, the upregulated genes were 5, 196, and 20, respectively. The downregulated genes were 2, 42, and 5, respectively. In the LAF group, 199, 694, and 46 DEGs were identified between 42 d of age vs. 56 d of age, 42 d of age vs. 70 d of age, and 56 d of age vs. 70 d of age, respectively. Among these DEGs, the upregulated genes were 54, 455, and 43, respectively. The downregulated genes were 145, 239, and 3, respectively.

#### GO and KEGG enrichment analysis of DEGs

Based on the identification of DEGs for each comparison group, GO and KEGG functional enrichment analyses were performed. At 42, 56, and 70 d of age, since the EAF group has only single-digit differential genes compared to the LAF group, there is not much gene enrichment in the pathway, so we do not describe it too much. There were a relatively large number of DEGs between the two treatment groups of lambs at 42 d of age and 70 d of age. The functional enrichment analysis of these DEGs between 42 and 70 d of age can reflect the changes in rumen epithelial genes of lambs induced by age under different treatment conditions. Therefore, we conducted GO and KEGG enrichment analyses on the DEGs at 42 d of age and 70 d of age. The GO analysis revealed that DEGs identified from the ‌42 d vs. 70 d comparison ‌in the EAF group were significantly enriched in “negative regulation of transmembrane receptor protein serine/threonine kinase signaling pathway” (Supplementary Figure S5A). However, GO pathway analysis revealed enrichment of DEGs from the LAF group (42 d vs 70 d) in “regulation of transmembrane receptor protein serine/threonine kinase signaling pathway” (Supplementary Figure S5B). The KEGG enrichment analysis indicated that there were no significantly enriched pathways for the DEGs in the EAF group at both 42 d of age and 70 d of age (Supplementary Figure S5C). The DEGs in the LAF group at 42 d of age and 70 d of age were enriched in pathways such as “Base excision repair,” “Calcium signaling pathway,” “Apoptosis” as well as pathways related to the immune system (Supplementary Figure S5D).

#### qRT-PCR validation of rumen epithelial genes

As presented in Supplementary Table S3, we selected six genes to verify the results of the transcriptomics. At 42, 56, and 70 d of age, there were no significant differences (*P* > 0.05) in the relative expression levels of *HMGCS2*, *HMGCL*, *ACAT1*, *BDH1*, *SLC9A3*, and *SLC9A2* between the EAF group and the LAF group. In both the EAF and LAF groups, there were no significant differences (*P* > 0.05) in the relative expression levels of *HMGCS2*, *ACAT1*, *BDH1*, *SLC9A3*, and *SLC9A2* across different ages. In the LAF group, the relative expression level of *HMGCL* at 42 d of age was significantly higher than that at 56 and 70 d of age (*P* < 0.05). The transcriptome sequencing results aligned with the findings of qRT-PCR, as presented in Supplementary Tables S4 to S6, thereby confirming the reliability of the transcriptome data.

#### Correlation analysis of rumen development indicators, differential bacteria, and differential genes in the EAF and LAF groups at 42 d of age

**Figure 3.**
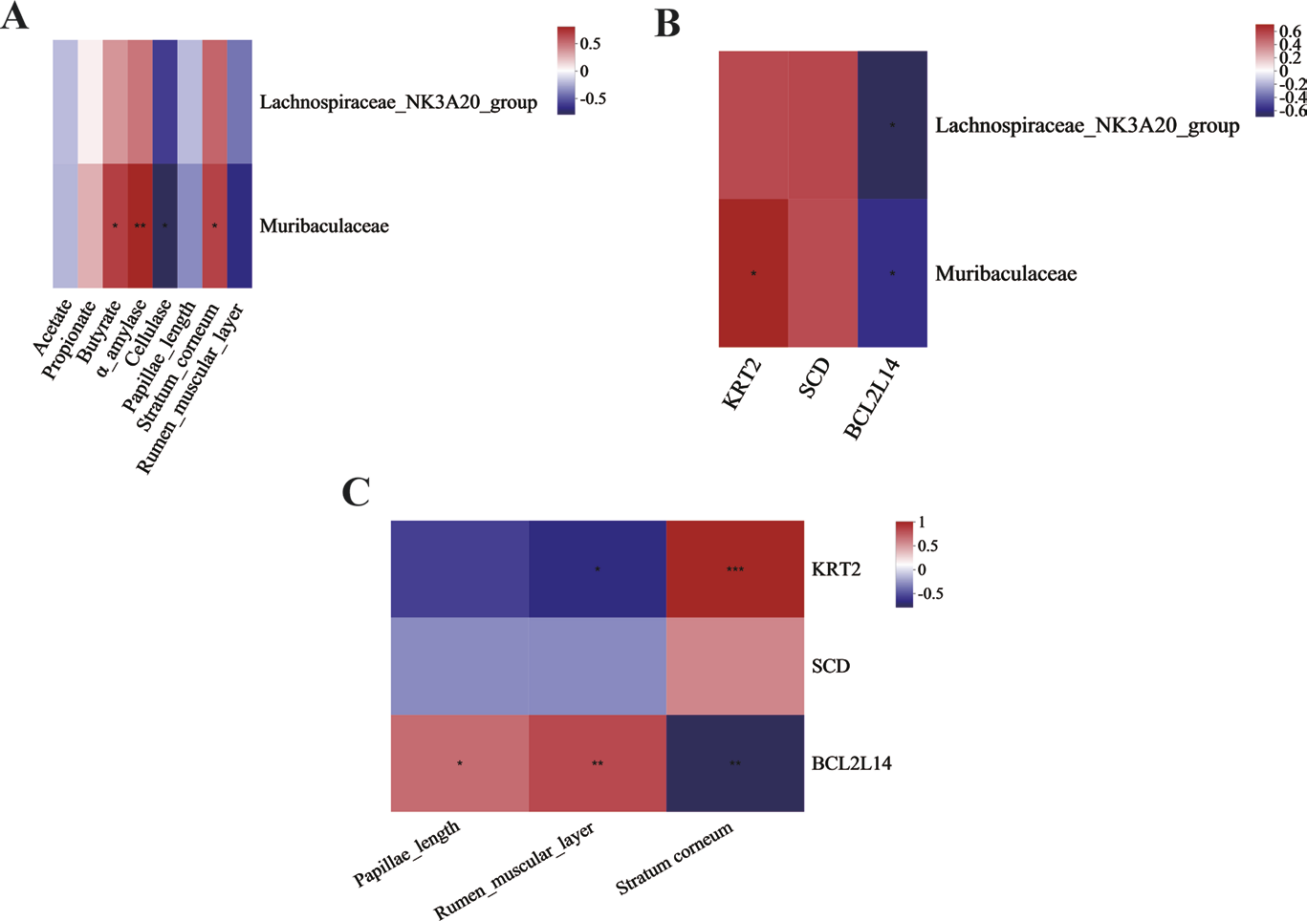
Spearman correlation analysis of rumen development indicators, differential bacteria, and differential genes. *P ≤ 0.05, **P ≤ 0.01, ***P ≤ 0.001.

The correlation analysis of rumen fermentation parameters, rumen development indicators, and differential bacteria reveals a positive correlation between the *Muribaculaceae* and butyrate (*P* < 0.05), α-amylase (*P* < 0.01), and rumen SC (*P* < 0.05) (Figure 3A). The correlation analysis of rumen differential genes and differential bacteria reveals a positive correlation between *KRT2* and *Muribaculaceae* (*P* < 0.05) (Figure 3B). Furthermore, *BCL2L14* exhibited a significant negative correlation with both *Lachnospiraceae_NK3A20_group* (*P* < 0.05) and *Muribaculaceae* (*P* < 0.05) (Figure 3B). The correlation analysis of rumen development indicators and differential genes reveals a positive correlation between the *KRT2* and rumen SC thickness (*P* < 0.001), but a negative correlation with rumen muscular layer thickness (*P* < 0.05) (Figure 3C). Moreover, significant positive correlations were identified between *BCL2L14* and both rumen papillae length (*P* < 0.05) and muscular layer thickness (*P* < 0.01), but a negative correlation with rumen SC thickness (*P* < 0.01) (Figure 3C).

## Discussion

Forage serves as a crucial feed source for young ruminants, significantly contributing to their health and growth. Xiao et al. (2023) recommended starting to feed high-quality hay to calves from the second week after birth, rather than immediately after their birth, based on observations of rumination development, feed intake, nutrient digestibility, and growth performance. Compared with calves, the gastrointestinal tract volume of lambs is smaller and DMI is different, so the optimal forage supplementation time still needs further investigation. In our study, starting hay provision at 14 d of age improved BW, ADG, and total DMI at 42 d of age compared to adding hay at 42 d of age, but had no effect after 42 d of age. It implies that consuming hay at 14 d of life may improve the total DMI and growth performance of lambs, but has no long-term effect on the total DMI and growth performance. Hosseini et al. (2016) found that adding 15% of chopped alfalfa hay to the starter feed for 2-wk-old calves could increase their starter feed intake and ADG. Similarly, we found that although the intake of starter feed between the EAF and LAF groups did not show a statistically significant difference at 21, 28, 35, and 42 d of age, there is a large gap between the data (98.89 vs. 74.16, 187.73 vs. 146.83, 256.54 vs. 204.94, 332.96 vs. 297.08). In addition, we found no significant difference in alfalfa hay intake between the EAF and LAF groups when the LAF lambs started to consume alfalfa hay at 42 d of age. After the LAF group lambs began to consume alfalfa hay at the age of 42 d, alfalfa hay intake increased rapidly, which may be related to the development of their digestive system, their curiosity, and desire to explore roughage.

Healthy development of the rumen is essential to ensure ruminant growth. It is generally believed that feeding forage to young ruminants before weaning is beneficial to the healthy development of the rumen (Yang et al., 2015; Nemati et al., 2016). However, there is a notable lack of research investigating the impact of forage supplementation timing on rumen development in preweaning lambs. In this study, feed plaques were observed on the rumen mucosa in the LAF group at 42 d of age, whereas no such plaques were seen in the EAF group at 42 d of age. Plaques cause the clustering of rumen papillae, reducing the absorptive area of the rumen epithelium for nutrients, which is detrimental to rumen health. Reducing the formation of feed plaques is beneficial for increasing the absorption area of the rumen epithelium, slowing the accumulation of VFA, and maintaining an optimal pH level in the rumen. The above results suggest that early supplementation with alfalfa hay can reduce the occurrence of rumen feed plaques, allowing the rumen to maintain healthy development. Furthermore, we found that the EAF lambs exhibited a lower thickness of rumen epithelial SC compared to the LAF lambs at 42 d of age. Gaebel et al. (1987) showed that the number of SC epithelial cells in the rumen is significantly influenced by the diet composition. The rumen epithelium undergoes constant renewal due to the friction with the chyme in the rumen, leading to the shedding of SC cells. Nevertheless, when the scavenging capacity of the diet on the rumen epithelial SC diminishes, the rumen squamous epithelial cells produce a hard keratin layer, leading to parakeratosis (Hinders and Owen, 1965). A thick SC can decrease the absorption of VFA, hinder blood flow of the rumen epithelium, and cause degeneration and loss of rumen papillae (Beharka et al., 1998). Early supplementation with alfalfa hay enriched the fiber content of the rumen digest, which enables the continuous contact with the rumen epithelium to remove keratin or dead epithelial cells. Consequently, the rumen epithelium maintains an appropriate degree of keratinization and integrity. In addition, in current study, it was found that at 42 d of age, the rumen papillae length and muscle layer thickness in the EAF group was significantly higher than that in the LAF group, indicating that early supplementary feeding of alfalfa hay to preweaning lambs can increase the rumen development. The elongation of rumen papillae facilitates the enlargement of the surface area available for nutrient absorption by the rumen epithelium, while an augmented thickness of the rumen muscle layer may enhance rumination in animals (Tamate et al., 1962). Greater rumen papillae length and greater rumen muscle layer thickness can eventually lead to better performance in a lamb production. However, at 56 and 70 d of age, there were no significant differences in growth performance, rumen fermentation parameters, and tissue morphology indicators between the EAF and LAF groups. These results indicated that this advantage gradually diminishes as the lambs grow older, and the effect of feed characteristics on rumen development may play a more important role. Furthermore, our findings revealed no differences in pH and VFA levels between the treatment groups during the overall period. Consistent with our findings, Wu et al. (2017) and Xiao et al. (2023) found that the age at which young ruminants voluntarily initiate forage consumption does not affect rumen pH and fermentation parameters. This may be attributed to the relatively small and insufficient hay intake, which is insufficient to elicit significant changes in rumen fermentation parameters (Xiao et al., 2023). In this study, at 42 d of age, the α-amylase activity in the EAF group was lower than that in the LAF group. The activity of rumen enzymes is strongly related to the abundance and metabolism of microorganisms within the rumen. These results suggested that early supplementation with alfalfa hay may affect the activity and abundance of rumen amylolysis bacteria.

To further understand the effect of alfalfa hay addition time, in rumen microbiota were observed between the EAF group and the LAF group. Our results showed that the addition time of alfalfa hay mainly influenced the microbial composition at 42 d of age. Specifically, the relative abundances of *Lachnospiraceae*_*NK3A20*_group and norank_f_*Muribaculaceae* increased significantly in the LAF group. The *Lachnospiraceae*_*NK3A20* is a genus within the Firmicutes phylum in the rumen, capable of degrading plant cellulose and hemicellulose, which are difficult for the host to digest (Wang et al., 2019). Wang (2018) reported that feeding alfalfa saponins to calves significantly reduced the relative abundance of *Lachnospiraceae*_*NK3A20*_group. Consequently, we speculate that the higher relative abundance of *Lachnospiraceae*_*NK3A20*_group in the LAF lambs compared to EAF lambs at 42 d of age may be attributed to the saponins contained in alfalfa. The Norank_f_*Muribaculaceae* is an important VFA-producing bacteria belonging to the phylum Bacteroidota (Hou et al., 2022). Meanwhile, we observed a significant positive correlation between α-amylase and *Muribaculaceae*. Furthermore, in our study, there was no significant difference in the microbial composition between EAF and LAF lambs after both groups were exposed to alfalfa hay. Consistent with our findings, Yang et al. (2018) reported that alfalfa hay intervention changes the composition of the rumen microbial community in lambs during early life. However, when both groups were exposed to alfalfa hay, there was no significant difference in the microbial composition of the rumen between treatments. Compared with the timing of hay supply, age is a crucial factor in the colonization of rumen microbiota. Thus, we explored the colonization process of the bacterial biomarkers under different feeding schemes. In the present study, the bacterial biomarkers specific to the EAF group at 42 d of age were *Eubacterium*_*coprostanoligenes*_group and *Prevotellaceae*_*YAB2003*_group. Meanwhile, a biomarker specific to the LAF group at 42 d of age was *Lachnospiraceae*_*NK3A20*_group. Interestingly, *Eubacterium*_*coprostanoligenes*_group emerged as a specific biomarker for the LAF group at 56 d of age. And, *Succiniclasticum* was mainly enriched in the EAF group at 56 d of age. Moreover, in the LAF group, *Succiniclasticum* was mainly enriched at 70 d of age. *Eubacterium*_*coprostanoligenes* is a cellulolytic fiber-degrading bacteria (Ma et al, 2021). *Prevotellaceae*_*YAB2003*_group are amylolytic and proteolytic bacteria that can produce propionate (Matsui et al., 2000). *Succiniclasticum* is a saccharolytic bacteria that produces acetate and lactate (Yang et al., 2018). In addition, we also found that three specific biomarkers were only enriched in the EAF group at 56 d of age, including *Acetitomaculum* (butyrate producers) (Shen, 2022), *Ruminococcus*_*gauvreauii*_group (organic acid-producing bacteria relating to fiber digestion) (Yang et al., 2018), and norank_f_*Muribaculaceae*. These results suggested that early supplementation with alfalfa can regulate the establishment of rumen microbiota.

To understand the temporal dynamics of the ruminal epithelium transcriptome in response to alfalfa hay addition time. We investigated the differences in rumen epithelial gene expression and its changes with age under different feeding regimens. For the diet effect, only three genes (*BCL2L14*, *KRT2*, and *SCD*) were differentially expressed between the EAF and LAF groups at 42 d of age, and the expression of the *KRT2* gene was significantly reduced in the EAF group. The *KRT2* gene is associated with keratinocyte differentiation and plays a key role in the formation of SC (Bragulla and Homberger, 2009). In our study, a positive correlation was identified between the *KRT2* and rumen SC thickness. Thus, we speculated that the excessive expression level of *KRT2* may lead to excessive thickness of the rumen SC and ruminal parakeratosis. This may affect the absorption function and metabolic activity of the rumen epithelium, thus affecting its normal physiological function (Yuan et al., 2022). Besides the physical removal of keratinocytes through friction between alfalfa hay and the rumen epithelium, it may also inhibit the expression of genes related to keratinocyte proliferation (specifically the *KRT2* gene) through certain metabolites. Through the correlation heatmap, we found that *Muribaculaceae* is significantly positively correlated with *KRT2*. This suggests that the expression of *KRT2* may be regulated by *Muribaculaceae. BCL2L14* is related to the regulation of the apoptotic process. The expression of *BCL2L14* in the EAF group was significantly higher than that in the LAF group, indicating that early supplementary feeding of alfalfa hay helps maintain the homeostasis of rumen epithelial cells by removing damaged or excess cells and preventing the accumulation of abnormal cells. Meanwhile, in this study, we found that there were significant positive correlations between *BCL2L14* and both rumen papillae length and muscular layer thickness, but a negative correlation with rumen SC thickness. In addition, we also found that *BCL2L14* exhibited a significant negative correlation with *Muribaculaceae*, and the abundance of *Muribaculaceae* was positively correlated with the thickness of the rumen SC. Therefore, the *Muribaculaceae* may affect the development of the rumen by regulating the expression of *KRT2* and *BCL2L14*, but the underlying regulatory mechanism warrants further in-depth investigation. *SCD* is related to unsaturated fatty acid metabolism. Therefore, the decreased expression of the *SCD* gene in the EAF group compared to the LAF group suggests that early supplementary feeding of alfalfa hay may affect the metabolism of unsaturated fatty acids in the rumen epithelium. At 56 d of age, the expression of the *CSMD2* gene in the rumen epithelium of lambs was upregulated in the EAF group compared to the LAF group. At 70 d of age, the expression of the *SNCA* gene in the rumen epithelium of lambs was downregulated in the EAF group compared to the LAF group. However, no significant differences in rumen epithelial tissue morphological indices were observed between the EAF and LAF groups at 56 and 70 d of age. Therefore, further in-depth investigations are warranted to elucidate the potential roles of *CSMD2* and *SNCA* in rumen development.

## Conclusion

Compared with adding alfalfa hay at 42 d of age, adding alfalfa hay at 14 d of age can increase the ADG and total DMI of lambs. The improvement of lamb production performance may be related to the early supplementary feeding of alfalfa hay promoting the healthy development of the rumen. However, this advantage gradually diminishes as the lambs grow older. Early supplementation of alfalfa hay promotes rumen development through the regulation of *KRT2* and *BCL2L14* gene expression.

## Supplementary Material

skaf227_suppl_Supplementary_Materials_1

## Data Availability

The rumen microbial sequencing data and rumen epithelial transcriptome data of this study are available in the NCBI SRA database with the BioProject ID: PRJNA1020448 and ID: PRJNA1021964, respectively.

## Abbreviations

ACAT1: acetyl-CoA acetyltransferase 1
ADG: average daily gain
BCL2L14: solute carrier family 2 like 14
BDH1: 3-hydroxybutyrate dehydrogenase 1
BW: body weight
DEGs: differentially expressed genes
DMI: dry matter intake
EAF: early alfalfa feeding group
FDR: false discovery rate
GAPDH: glyceraldehyde-3-phosphate dehydrogenase
GO: Gene Ontology
HMGCL: 3-hydroxymethyl-3-methylglutaryl-CoA lyase
HMGCS2: 3-hydroay-3-methylglutaryl-CoA synthase 2
KEGG: Kyoto Encyclopedia of Genes and Genomes
KRT2: keratin 2
LAF: late alfalfa feeding group
MR: milk replacer
OTUs: operational taxonomic units
RIN: RNA integrity number
SB: stratum basale
SC: stratum corneum
SCD: stearoyl-CoA desaturase
SG: stratum granulosum
SLC9A2: solute carrier family 9 member A2
SLC9A3: solute carrier family 9 member A3
SS: stratum spinosum
VFA: volatile fatty acids
