# Supplementary material for: Effects and mechanism of the timing of alfalfa hay supplementation on rumen development of Hu lambs
Source: J Anim Sci. 2025 Aug 23;103:skaf227. doi: 10.1093/jas/skaf227 (PMC12391868; doi:10.1093/jas/skaf227)
Supplement: skaf227_suppl_Supplementary_Materials_1 [file skaf227_suppl_supplementary_materials_1.docx]

**Running head: Early addition of alfalfa boosts rumen development**

**Effects and mechanism of the timing of alfalfa hay supplementation on rumen development of Hu lambs**

Kenan Li^1,2,^ Haidong Du^1^, Wenliang Guo^1^, Meila Na^1^, Renhua Na^1*^

^1^ College of Animal Science, Inner Mongolia Agricultural University, Hohhot 010018, China.

^2^ Institute of Grassland Research of Chinese Academy of Agricultural Sciences, Hohhot 010010, China.

*** Correspondence author: narenhualaoshi@163.com

**Table S1.** Nutrient components of milk replacer, starter pellets, and alfalfa hay (air-dry basis, %)

| Items | Starter pellets | Milk replacer | Alfalfa hay |
| --- | --- | --- | --- |
| Ingredients | | | |
| Corn | 40.00 | - | - |
| DDGS | 3.00 | - | - |
| Soybean meal | 26.00 | - | - |
| Wheat bran | 13.00 | - | - |
| Corn germ meal | 6.00 | - | - |
| Soybean hulls | 4.00 | - | - |
| Extruded soybean | 4.00 | - | - |
| Limestone | 2.00 | - | - |
| CaHPO_4_ | 0.50 | - | - |
| NaCl | 0.50 | - | - |
| Premix^1^ | 1.00 | - | - |
| Total | 100.00 | - | - |
| Chemical composition | | | |
| DM | 89.70 | 94.45 | 86.63 |
| CP | 23.98 | 20.88 | 15.20 |
| EE | 4.86 | 11.08 | 2.23 |
| NDF | 22.46 | - | 55.96 |
| ADF | 8.10 | - | 43.07 |
| Ash | 7.48 | 2.59 | 7.09 |
| Ca | 0.98 | 1.05 | 1.35 |
| P | 0.72 | 0.68 | 0.16 |
| Metabolizable energy (MJ/Kg)^2^ | 11.67 | 14.16 | 7.96 |

DDGS: distiller’s dried grains with solubles. DM: dry matter; CP: crude protein; EE: ether extract; NDF: neutral detergent fibers; ADF: acid detergent fiber; Ash: crude ash.

^1^Contained per kilogram of supplement: vitamin A, 800,000 IU; vitamin D3, 30,000 IU; vitamin E, 3000 mg; Cu, 0.8 g; Fe, 4 g; Mn, 4 g; Zn, 5 g; I, 70 mg; Se, 20 mg; Co, 40 mg.

^2^Nutrient levels were all measured except the Metabolizable energy. The calculation method of Metabolizable energy refers to the method of Nutrient Requirements of Meat-type Sheep and Goat (NY/T 816-2021).

**Table S2.** The primer sequencings designed and Amplification conditions for qRT-PCR

| Genes | Forward primer sequence (5' to 3') | Reverse primer sequence (5' to 3') | Amplification conditions |
| --- | --- | --- | --- |
| HMGCL | GCTCCACGAGACGGACTACAA | CTCAGAGGCGGCTCCAAAGAT | 30 s at 95°C followed by 40 cycles composed of 5 s at 95°C, 30 s at 55°C |
| HMGCS2 | TACCTGGAGCGAGTGGATGA | GGCGAGTCATCTGGATCTGG | 30 s at 95°C followed by 40 cycles composed of 15 s at 95°C, 60 s at 60°C |
| BDH1 | GAGAAGGAAACGGCGGTAG | AAAAGGCAGAATGGTCAGG | 30 s at 95°C followed by 40 cycles composed of 5 s at 95°C, 34 s at 60°C |
| ACAT1 | TGGCCTCTCAGAATCTTATGTGT | CATACGGTGTTGCTCCTCTGTT | 30 s at 95°C followed by 40 cycles composed of 5 s at 95°C, 30 s at 60°C |
| GAPDH | GGGTCATCATCTCTGCACCT | GGTCATAAGTCCCTCCACGA | 30 s at 95°C followed by 40 cycles composed of 5 s at 95°C, 31 s at 60°C |
| SLC9A3 | AGCTACGTGGCCGAGGG | AGACAGAGGCCTCCACGGT |  |
| SLC9A2 | TTGGAGAGTCCCTGCTGAAC | GGCCGTGATGTAGGACAAAT |  |

HMGCS2: 3-hydroay-3-methylglutaryl-CoA synthase 2; BDH1: 3-hydroxybutyrate dehydrogenase 1; HMGCL: 3-Hydroxymethyl-3-methylglutaryl-CoA lyase; ACAT1: acetyl-CoA acetyltransferase 1; SLC9A3: solute carrier family 9 member A3; SLC9A2: solute carrier family 9 member A2; GAPDH: glyceraldehyde-3-phosphate dehydrogenase.

**Table S3.** The relative expressions of HMGCS2, HMGCL, ACAT1, BDH1, SLC9A3 and SLC9A2 were quantified by qRT-PCR

| Items | Diet^1^ | | Days of age | | |  | *P* for diets | | | *P* for age |
| --- | --- | --- | --- | --- | --- | --- | --- | --- | --- | --- |
|  |  |  | 42 d | 56 d | 70 d |  | 42 d | 56 d | 70 d |  |
| HMGCS2 | EAF | 0.90±0.37 | | 0.97±0.57 | 0.97±0.69 | | 0.53 | 0.27 | 0.78 | 0.97 |
|  | LAF | 0.71±0.54 | | 0.60±0.38 | 1.09±0.43 | |  |  |  | 0.29 |
| HMGCL | EAF | 1.06±0.43 | | 1.13±0.33 | 1.37±0.35 | | 0.19 | 0.30 | 0.99 | 0.36 |
|  | LAF | 0.75±0.34B | | 0.90±0.39A | 1.37±0.33A | |  |  |  | 0.02 |
| ACAT1 | EAF | 1.08±0.49 | | 1.11±0.41 | 1.63±0.25 | | 0.63 | 0.49 | 0.96 | 0.09 |
|  | LAF | 0.97±0.23 | | 1.17±0.28 | 1.60±0.41 | |  |  |  | 0.06 |
| BDH1 | EAF | 1.08±0.53 | | 0.95±0.21 | 1.39±0.37 | | 0.32 | 0.41 | 0.77 | 0.21 |
|  | LAF | 0.78±0.45 | | 1.25±0.82 | 1.30±0.56 | |  |  |  | 0.32 |
| SLC9A3 | EAF | 0.97±0.31 | | 0.67±0.39 | 1.39±1.09 | | 0.10 | 0.93 | 0.70 | 0.24 |
|  | LAF | 0.62±0.32 | | 0.69±0.35 | 1.69±1.50 | |  |  |  | 0.11 |
| SLC9A2 | EAF | 1.09±0.52 | | 1.10±0.48 | 1.14±0.56 | | 0.10 | 0.23 | 0.95 | 0.71 |
|  | LAF | 0.85±0.65 | | 0.97±0.54 | 1.27±0.44 | |  |  |  | 0.09 |

^1^ EAF: early (at 14 d of age) feeding of alfalfa. LAF: late (at 42 d of age) feeding alfalfa.

HMGCS2: 3-hydroay-3-methylglutaryl-CoA synthase, HMGCL: 3-Hydroxymethyl-3-methylglutaryl-CoA lyase, BDH1: 3-hydroxybutyrate dehydrogenase 1, ACAT1: acetyl-CoA acetyltransferase 1, SLC9A3: solute carrier family 9 member A3, SLC9A2: solute carrier family 9 member A2.

The superscripts of the lowercase letters in the same column represent the differences between the EAF and LAF groups at the same age, and different lowercase letters indicate significant differences (*P*<0.05). The superscripts of the capital letters on the same line represent the differences among different ages within the same treatment group, and different capital letters indicate significant differences (*P*<0.05).

**Table S4.** Differentially expressed genes of rumen fatty acid metabolism in LAF_42 and EAF_42 groups

| Gene ID | Gene name | FC (LAF_42/EAF_42) | Log2FC (LAF_42/EAF_42) | Padjust |
| --- | --- | --- | --- | --- |
| ENSOARG00020001485 | HMGCS2 | 0.776 | -0.366 | 0.996 |
| ENSOARG00020013128 | HMGCL | 0.884 | -0.179 | 0.996 |
| ENSOARG00020011001 | ACAT1 | 0.831 | -0.266 | 0.996 |
| ENSOARG00020008942 | BDH1 | 0.849 | -0.237 | 0.996 |
| ENSOARG00020014019 | SLC9A3 | 1.059 | 0.083 | 0.996 |
| ENSOARG00020002529 | SLC9A2 | 0.798 | -0.325 | 0.996 |

EAF_42: early-fed alfalfa hay lambs at 42 d of age. FC: fold change.

**Table S5.** Differentially expressed genes of rumen fatty acid metabolism in LAF_56 and EAF_56 groups

| Gene ID | Gene name | FC (LAF_56/EAF_56) | Log2FC (LAF_56/EAF_56) | Padjust |
| --- | --- | --- | --- | --- |
| ENSOARG00020001485 | HMGCS2 | 0.885 | -0.176 | 1.00 |
| ENSOARG00020013128 | HMGCL | 1.002 | 0.003 | 1.00 |
| ENSOARG00020011001 | ACAT1 | 0.996 | -0.006 | 1.00 |
| ENSOARG00020008942 | BDH1 | 0.965 | -0.051 | 1.00 |
| ENSOARG00020014019 | SLC9A3 | 0.788 | -0.344 | 1.00 |
| ENSOARG00020002529 | SLC9A2 | 1.114 | 0.156 | 1.00 |

EAF_56: early-fed alfalfa hay lambs at 56 d of age. FC: fold change.

**Table S6.** Differentially expressed genes of rumen fatty acid metabolism in LAF_70 and EAF_70 groups

| Gene ID | Gene name | FC (LAF_70/EAF_70) | Log2FC (LAF_70/EAF_70) | Padjust |
| --- | --- | --- | --- | --- |
| ENSOARG00020001485 | HMGCS2 | 0.915 | -0.128 | 1.00 |
| ENSOARG00020013128 | HMGCL | 0.928 | -0.107 | 1.00 |
| ENSOARG00020011001 | ACAT1 | 0.939 | -0.90 | 1.00 |
| ENSOARG00020008942 | BDH1 | 0.964 | -0.052 | 1.00 |
| ENSOARG00020014019 | SLC9A3 | 0.952 | -0.071 | 1.00 |
| ENSOARG00020002529 | SLC9A2 | 0.905 | -0.144 | 1.00 |

EAF_70: early-fed alfalfa hay lambs at 70 d of age. FC: fold change.

**
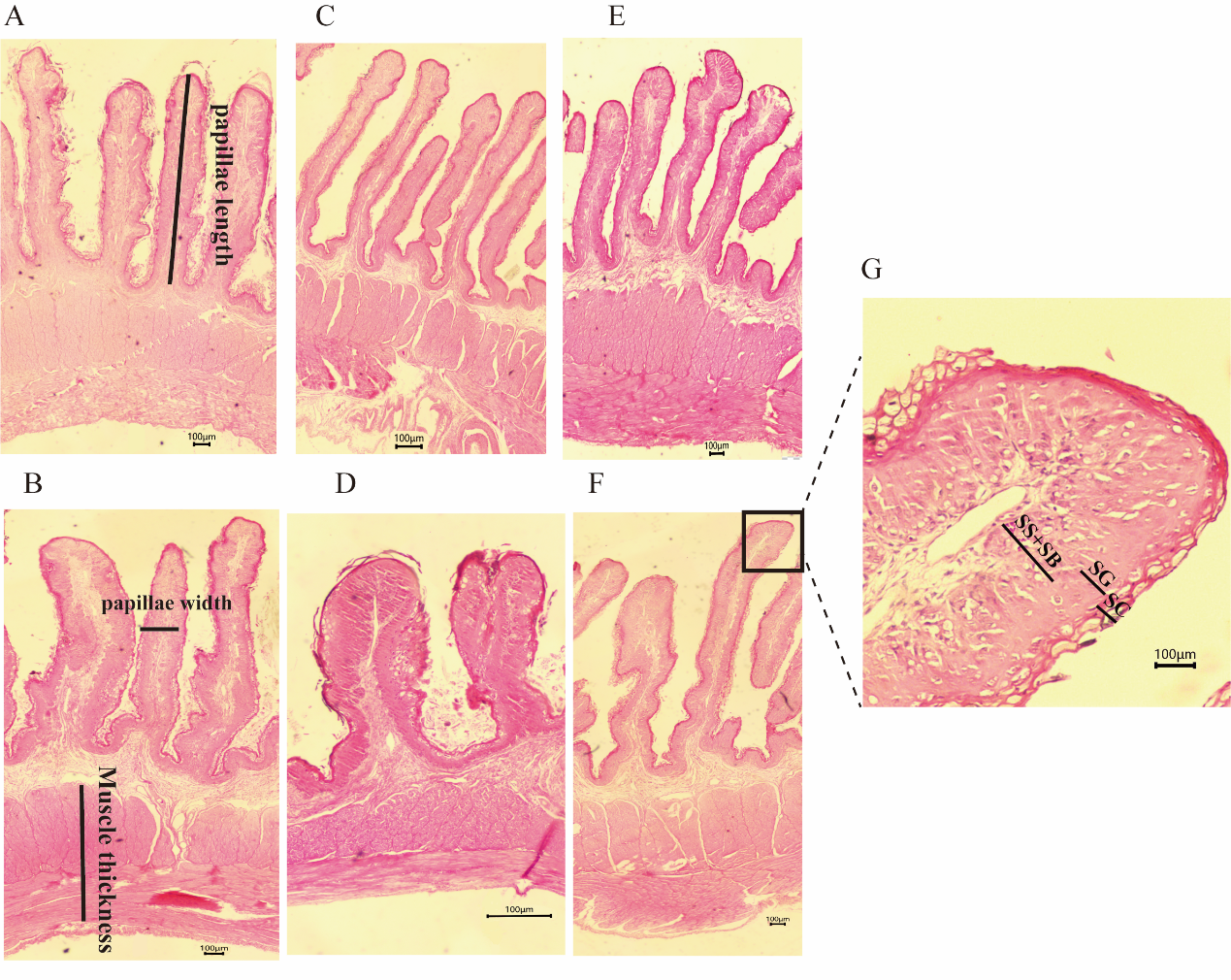
**

**Fig. S1.** Representative micrograph of the rumen epithelium of the lambs at different ages of development and in response to different dietary treatments. A: at 42 d of age, early-fed alfalfa hay (EAF); B: at 42 d of age, late-fed alfalfa hay (LAF); C: the EAF lambs at d 56; D: the LAF lambs at d 56; E: the EAF lambs at d 70; F: the LAF lambs at d 70. SC = stratum corneum; SG = stratum granulosum; SS = stratum spinosum; SB = stratum basale.

**
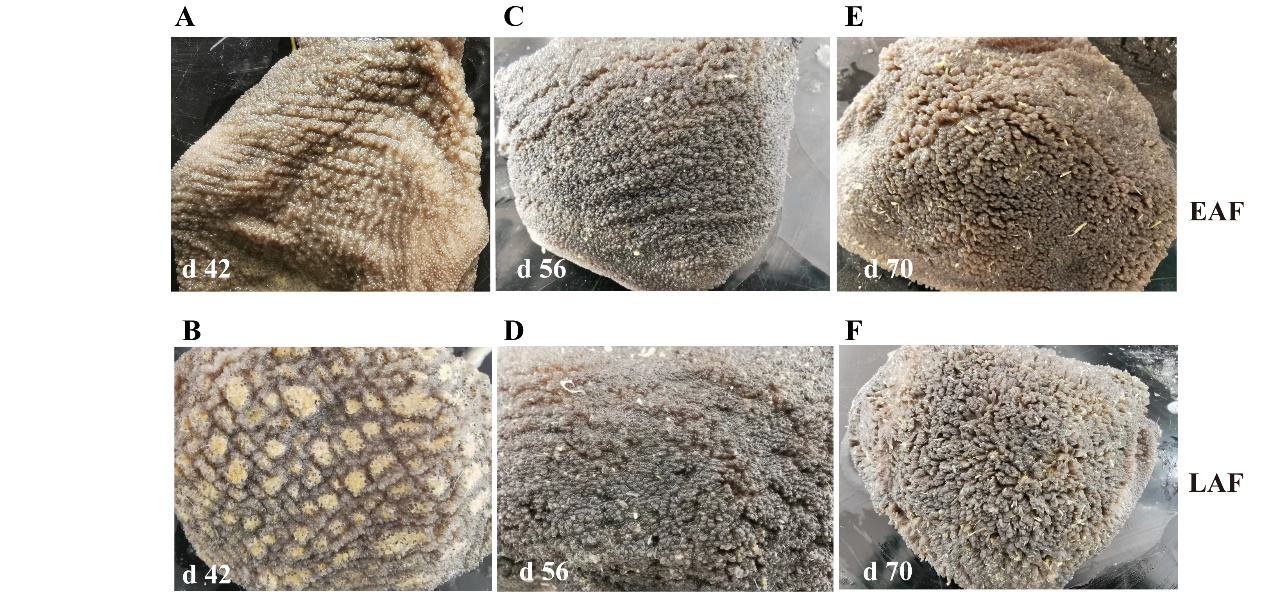
**

**Fig. S2** Representative images of the rumen epithelium of the lambs at different ages of development and in response to different dietary treatments. Early-fed alfalfa hay (EAF) lambs at 14 d of age **(A**); late-fed alfalfa hay (LAF) lambs at 42 d of age; **C**: the EAF lambs at 56 d of age **(B**); the LAF lambs at 56 d of age **(D**); **E**: the EAF lambs at 70 d of age; the LAF lambs at 70 d of age **(F**).


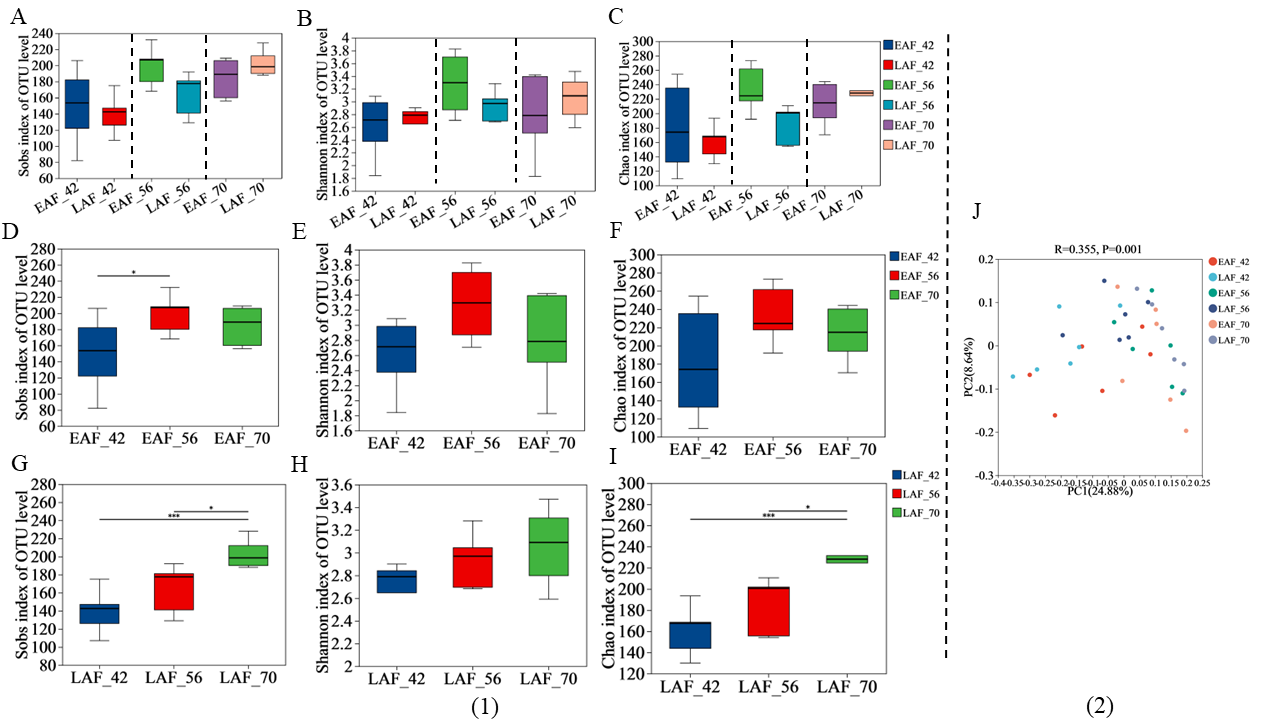


**Fig. S3** Alpha and beta diversity of rumen microbiota in response to the timing of hay supply and age in pre-weaning lambs. (1) Alpha diversity (based on the Sobs, Shannon, and Chao indices) in the rumen microbial community was analyzed using the Kruskal-Wallis test and a post-hoc Tukey-kramer multiple comparison, and the FDR method was used for *P* value correction. Alpha diversity of rumen microbiota between EAF and LAF lambs at 42, 56 and 70 d of age **(A**, **B**, **C**). Microbial alpha diversities of EAF group at 42, 56 and 70 d of age (**D**, **E**, **F**). Microbial alpha diversities of LAF group at 42, 56 and 70 d of age (**G**, **H**, **I**). * *P*≤0.05, ** *P*≤0.01, *** *P*≤0.001. (2) The principal coordinate analysis (PCoA) was performed based on unweighted UniFrac distances (**J**). EAF: early (at 14 d of age) feeding of alfalfa. LAF: late (at 42 d of age) feeding alfalfa.

**
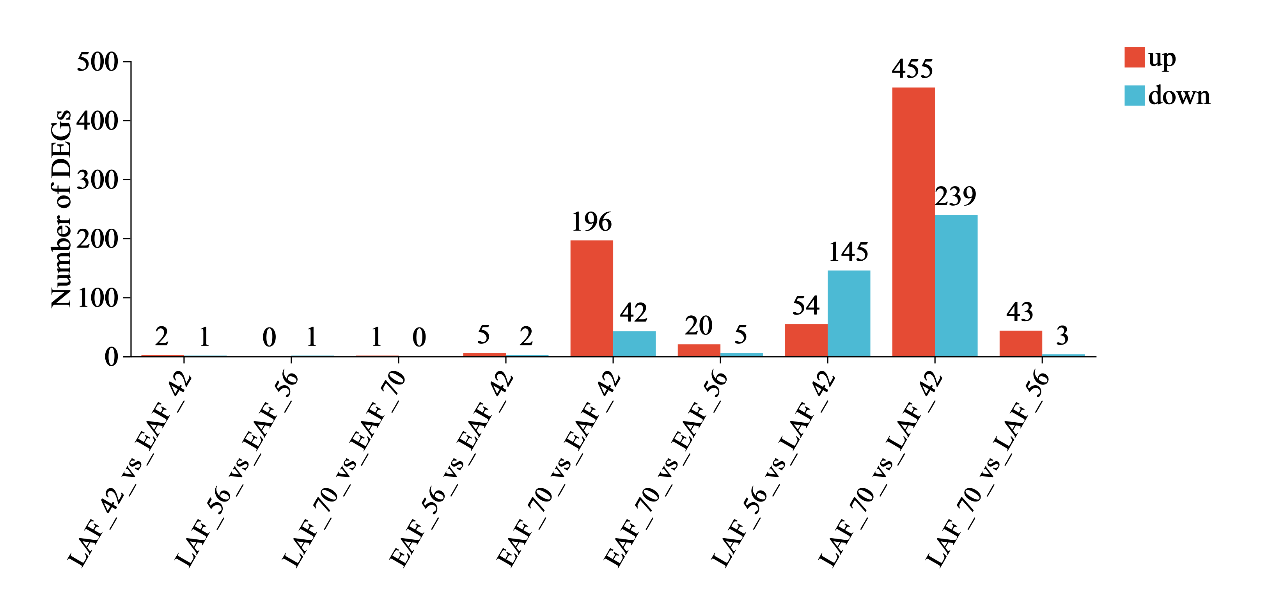
**

**Fig. S4** Differential gene quantity statistics. EAF= early (at 14 d of age) feeding of alfalfa. LAF= late (at 42 d of age) feeding of alfalfa.

**
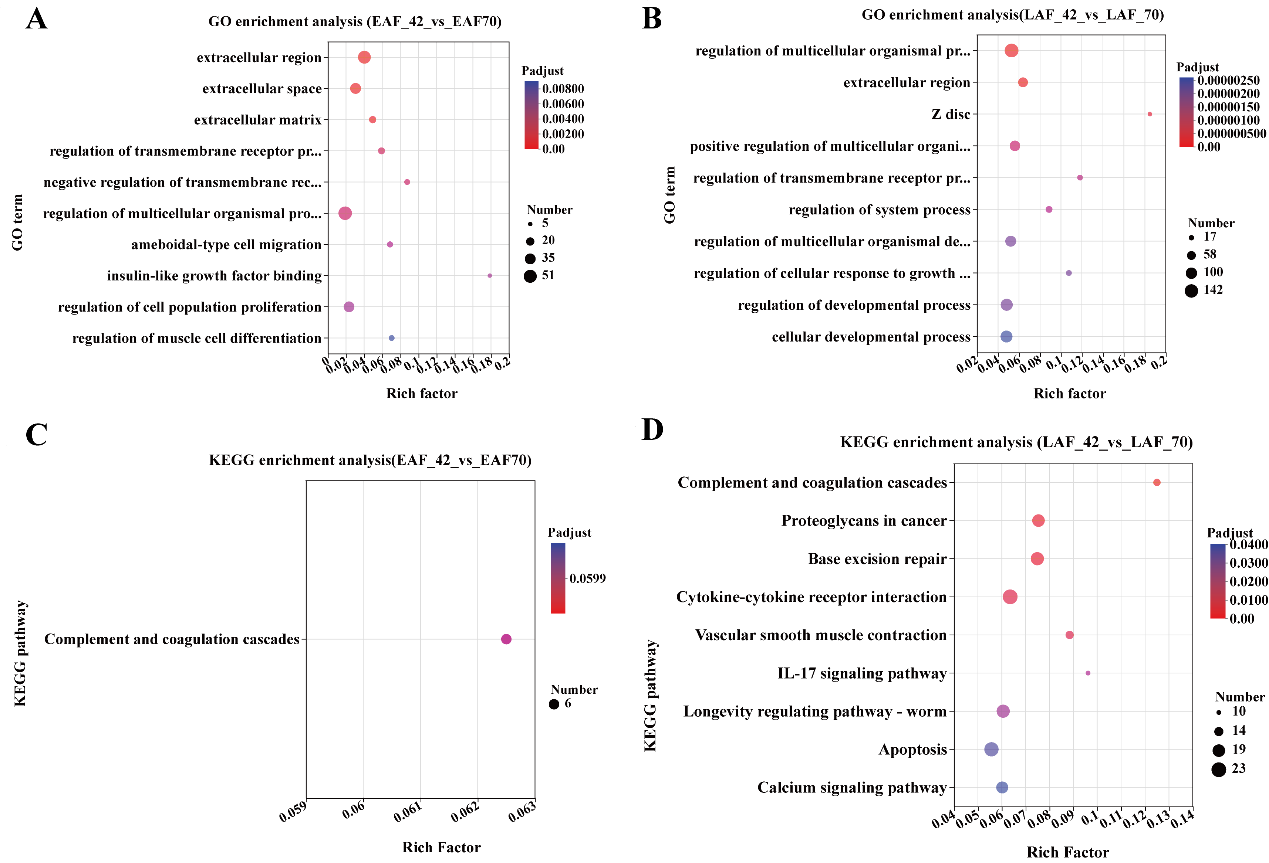
**

**Fig. S5** Polygenic set Bubble charts of GO and KEGG enrichment of differentially expressed genes (DEGs). **A**: In the EAF group, the top 10 GO items for DEGs comparing 42 d of age with 70 d of age; **B**: In the LAF group, the top 10 GO items for DEGs comparing 42 d of age with 70 d of age; **C**: In the EAF group, the top 10 KEGG items for DEGs comparing 42 d of age with 70 d of age; **D**: In the LAF group, the top 10 KEGG items for DEGs comparing 42 d of age with 70 d of age. The size of the point indicates the numbers of DEGs, the color of point indicates the false discovery rate (FDR, Padjust), the abscissa indicates the rich factor, and ordinate indicates the GO items or KEGG pathways. EAF: early (at 14 d of age) feeding of alfalfa hay; LAF: late (at 42 d of age) feeding alfalfa hay.
